# Biocompatible Alginate Film Crosslinked with Ca^2+^ and Zn^2+^ Possesses Antibacterial, Antiviral, and Anticancer Activities

**DOI:** 10.1021/acsomega.3c01935

**Published:** 2023-06-28

**Authors:** Alba Cano-Vicent, Alberto Tuñón-Molina, Hamid Bakshi, Roser Sabater i Serra, Iman M. Alfagih, Murtaza M. Tambuwala, Ángel Serrano-Aroca

**Affiliations:** † Biomaterials and Bioengineering Lab, Centro de Investigación Traslacional San Alberto Magno, Universidad Católica de Valencia San Vicente Mártir, Valencia 46001, Spain; ‡ Hormel Institute, University of Minnesota, Austin, Minnesota 55912, United States; § Centre for Biomaterials and Tissue Engineering, Universitat Politècnica de València, València 46022, Spain; ∥ Biomedical Research Networking Centre in Bioengineering, Biomaterials and Nanomedicine (CIBER-BBN), València 46022, Spain; ⊥ Department of Electrical Engineering, Universitat Politécnica de Valencia, Valencia 46022, Spain; # Department of Pharmaceutics, College of Pharmacy, King Saud University, Riyadh 4545, Saudi Arabia; ∇ Lincoln Medical School, University of Lincoln, Brayford Pool Campus, Lincoln LN6 7TS, U.K.

## Abstract

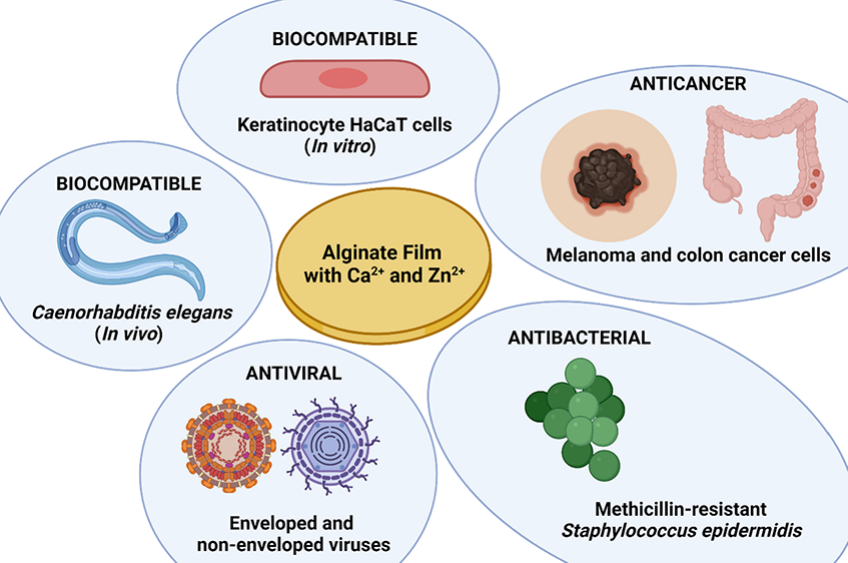

Alginate is a highly promising biopolymer due to its non-toxic and biodegradable properties. Alginate hydrogels are often fabricated by cross-linking sodium alginate with calcium cations and can be engineered with highly desirable enhanced physical and biological properties for biomedical applications. This study reports on the anticancer, antiviral, antibacterial, in vitro, and in vivo toxicity, water absorption, and compound release properties of an alginate hydrogel crosslinked with calcium and different amounts of zinc cations. The results showed that the calcium alginate hydrogel film crosslinked with the highest amount of zinc showed similar water sorption properties to those of calcium alginate and released a suitable amount of zinc to provide anticancer activity against melanoma and colon cancer cells and has antibacterial properties against methicillin-resistant *Staphylococcus epidermidis* and antiviral activity against enveloped and non-enveloped viruses. This film is non-toxic in both in vitro in keratinocyte HaCaT cells and in vivo in the *Caenorhabditis elegans* model, which renders it especially promising for biomedical applications.

## Introduction

Alginate is a promising biopolymer that can be produced either by microbial culture or from brown algae.^1^ It is also biodegradable and non-toxic, and its physical and biological properties can be enhanced by several strategies.^2^ As it dissolves in water, it is necessary to crosslink it with divalent cations to make it insoluble. Calcium chloride is among its most commonly used cross-linking compounds.^3^ The antiviral property of calcium alginate films against enveloped viruses has recently been discovered.^4^ This capacity is related to the negative charges of the hydrogel, which, when in contact with the proteins in the virus’s envelope, block its reproduction by inhibiting its entry into cells.^4^ The biocompatibility of this hydrophilic material has also been demonstrated in in vitro and in vivo assays.^5,6^

Adding bioactive compounds, such as zinc, silver, copper, or carbon-based materials, is among the strategies to enhance alginate hydrogel’s biological properties.^2^ In fact, antimicrobial scaffolds of 3D printed polylactic acid filled with bioactive alginate containing zinc cations were recently shown to have antibacterial and osteoinductive properties.^7^ The material containing zin cations showed antibacterial activity against Gram-positive methicillin-resistant *Staphylococcus epidermidis* (*S. epidermidis*; MRSE) and Gram-negative *Pseudomonas aeruginosa*, while the control material without zinc ions could not inhibit the two microbial species tested. Zinc cations are involved in cell growth and apoptosis by regulating transcription factors, enzymes, and growth factors.^8^ Zinc is one of the most important trace elements in the body, and it is capable of inducing cell proliferation, which is very desirable for biomedical applications.^9^ However, it is well-known that incorporating zinc cations can produce highly toxic materials.^10^

In this study, we hypothesized that a certain amount of zinc cations would provide a suitable zinc release to produce excellent antibacterial activity against MRSE, antiviral activity against enveloped (bacteriophage phi6) and non-enveloped (bacteriophage MS2) viruses, and anticancer properties against melanoma and colon cancer cells without compromising calcium alginate’s non-toxicity in human keratinocyte HaCaT cells and in vivo using the *Caenorhabditis elegans* (*C. elegans*) model. Since these materials are normally used in contact with water at body temperature, we also studied their equilibrium water sorption properties.

## Materials and Methods

### Materials

Sodium alginate (Sigma-Aldrich, USA), calcium chloride (≥93.0%, Sigma-Aldrich, USA), and zinc chloride (Sigma-Aldrich, USA).

### Fabrication of the Biofilm

Sodium alginate (0.25 g) was dissolved in 30 mL of distilled water by magnetic stirring for 1 h at 24 ± 0.5 °C. This mix was poured into a Petri dish and left for 24 h at room temperature, followed by 48 at 37 °C to form a film. Calcium chloride (5 g) was dissolved in 500 mL of distilled water by magnetic stirring for 15 min at 24 ± 0.5 °C to prepare the first crosslinking solution. The film was crosslinked with this solution for 1 h at 24 ± 0.5 °C. Next, the film was washed three times in a water solution. Different quantities of zinc chloride (0, 0.1, 0.01, and 0.001 g) were diluted in 500 mL of distilled water to prepare the second crosslinking solution. The films were then placed in different solutions of zinc for 2 h at 24 ± 0.5 °C, poured onto a Petri dish, and left for 24 h at room temperature, followed by 48 h at 37 °C to ensure complete drying. The films are hereafter referred to as Zn0 Zn0.1, Zn0.01, and Zn0.001 according to the weight of the zinc in each case. Disks (diameter 1 cm) were cut from each film and then sterilized by ultraviolet radiation for 1 h per side.

### Water Absorption Test

The films were dried at 60 °C for 48 h to constant weight, after which they were weighed and placed in 100 mL of distilled water and left in an oven at 37 °C. Thus, it was weighted at different water sorption times (30 min, 5 h, and 24 h). The water sorption (*h*) was calculated by the following formula 1:
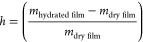
1where *m*_hydrated film_ is the weight of the swollen film for each time and *m*_dry film_ is the weight of the dry film.

### Zinc Release

The disks of the films were incubated individually with 600 μL of distilled water in a 48-well plate at 37 °C. The distilled water was replaced every 24 h for 5 days and at 10 days. The Zincon Assay (Sigma-Aldrich, St Louis, MO, USA) was used to quantify the amount of zinc released from the films. A microplate reader (Varioskan, Thermo Fisher) was used to read the absorbance at 570 nm.

### Electron Microscopy

The morphology of the hydrogels was analyzed by field emission scanning electron microscope (FESEM) (Zeiss ULTRA 55, Carl Zeiss Microscopy) with an accelerating voltage of 1.5 kV. The samples were lyophilized after swelling in liquid water at 37 °C for 24 h to constant weight and freezing at −80 °C overnight. The cross-section was observed in the lyophilized samples, which were previously immersed in liquid nitrogen and cryofractured for FESEM observation. Finally, the samples were coated with a carbon layer using a sputter coating (EM MED020, Leica). The percentages of Ca and Zn ions were obtained with an Energy Dispersive X-ray Spectrometry (EDX, X-Max N, Oxford Instruments) mounted on the Zeiss ULTRA 55 FESEM (accelerating voltage 15 kV).

### Fourier Transform Infrared Spectroscopy (FTIR)

FTIR (Bruker ALPHA II Compact FT-IR Spectrometer) was used to study the surface functional groups in a transmittance mode. All spectra were scanned at room temperature over the wave number range of 4000–400 cm^–1^ using 30 scans at a resolution of 2 cm^–1^.

### Toxicological In Vitro Study

Incubation of the sample disks was performed in a 6-well plate with DMEM (Biowest SAS, France) without fetal bovine serum (FBS) in humidified 5% CO_2_/95% ambient air for 72 h at 37 °C, following the ISO-10993 standard recommendations. This norm recommends a volume ratio of 3 cm^2^/mL for tube wall, slab, and small molded articles. The disk extracts were used immediately for the toxicological test. Non-tumorigenic immortalized human keratinocyte HaCaT cells provided by the Medical Research Institute Hospital La Fe, Valencia, Spain, were used in the studies. DMEM mixed with 10% FBS, 100 units/mL penicillin (Lonza, Belgium), and 100 mg/mL streptomycin (HyClone, GE Healthcare Life Sciences) was used for cell incubation at 37 °C and 5% CO_2_. The 3-[4,5-dimethylthiazol-2-yl]-2,5-diphenyl tetrazolium bromide (MTT) cytotoxicity assay was performed to study the effect of the extracts on cell viability. The cells were planted at 10^4^ cells/well onto a 96-well plate and incubated for 24 h at 37 °C. The medium in each well was then replaced with 100 μL of film extracts. For positive control, the medium was also replaced with 100 μL of the same medium used to produce the film extracts and with 100 μL of 1000 μM zinc chloride (≥97.0%, Sigma-Aldrich) toxic solution for the negative control.^10^ Finally, the medium was replaced with 5 mg/mL MTT for 3 h, so that 100 μL of dimethyl sulfoxide (Sigma-Aldrich) was added to the solution. The absorbance readings at 550 nm were performed on a microplate reader (Varioskan, Thermo Fisher).

### Proliferation Study

After the toxicological assay, no cytotoxic samples were used for the proliferation assay. In this case, the ISO-10993 standard recommendations were followed to extract the samples. The extracts were used immediately after incubation in humidified 5% CO_2_/95% ambient air for 72 h at 37 °C.

Non-tumorigenic immortalized human keratinocyte HaCaT cells provided by the Medical Research Institute Hospital La Fe, Valencia, Spain, were used for these studies. DMEM mixed with 0.5% FBS, 100 units/mL of penicillin (Lonza, Belgium), and 100 mg/mL of streptomycin (HyClone, GE Healthcare Life Sciences) were used for cell incubation. Cell viability was assayed by the MTT assay. The cells were planted at 5 × 10^3^ cells/well onto a 96-well plate and incubated for 24 h at 37 °C, after which the medium in each well was replaced with 100 μL of film extracts. The positive and negative controls were performed in the same way as in the cytotoxicity test.^17^ The medium was replaced with 100 μL of 15 ng/mL of EGF as proliferative control. The cells were incubated in humidified 5% CO_2_/95% ambient air for 72 and 96 h at 37 °C. The medium was then replaced with 5 mg/mL MTT in each well for 3 h. The formazan crystals were dissolved in 100 μL of dimethyl sulfoxide (Sigma-Aldrich) at room temperature, and the absorbance readings were performed at 550 nm on a microplate reader (Varioskan, Thermo Fisher).

### In Vivo Toxicity Tests

The *C. elegans* model was used to study the in vivo toxicity. The nematodes were provided by the *Caenorhabditis* Genetics Center (Minneapolis, MN, USA). An N2 strain was maintained and propagated on nematode growth medium (NGM) with OP50 *Escherichia coli* (*E. coli*) at 25 °C for the experiments on the synchronized worms. The plates with the nematodes were washed with 5 mL of distilled water. The tubes with the worms were centrifuged at 1300 rpm for 3 min, and the pellet was resuspended in 100 μL of distilled water and 700 μL of a 5% bleaching solution. The mixture was then vortexed every 2 min. This step was repeated five times. The tubes were centrifuged at 700 × *g* for 3 min, and the pellet with the worm eggs was resuspended with 800 μL of distilled water. This was carried out three times. In the last centrifuge, the pellet was resuspended in 100 μL of distilled water to transfer it to a NGM plate with OP50 *E. coli*. The plate with the nematode eggs was incubated for 72 h at 25 °C to obtain worms in an L1 staged population. After 72 h, the NGM plates were washed and centrifuged by the same procedure, and the pellet was resuspended in 3 mL of the potassium medium. The extraction of each sample was carried out by the same method as the in vitro toxicity, but the medium used was the autoclaved K medium (2.36 g potassium chloride and 3 g sodium chloride in 1 L of distilled water) and incubated 72 h at 25 °C. For the assay, a mixture with 62.5 μL of a 1:250 suspension of cholesterol (5 mg/mL in ethyl alcohol) in the sterile K medium, 62.5 μL of a 50× concentrated OP50 *E. coli* culture, 115 μL of potassium medium and 250 μL of the pertinent extract was carried out in a 48-well plate, and 50–100 worms were added to each well. Two controls were performed: a positive control (worms incubated with medium only and without extracts) and a negative (worms incubated with a zinc dilution). The plates were stamped with parafilm and placed in an orbital shaker at 25 °C and 120 rpm for 24 and 72 h. For the survival rate of *C. elegans*, the mixture incubation with the extracts and the worms was divided into 10 drops of 50 μL and placed under the microscope (Motic BA410E including Moticam 580 5.0MP). The living and dead worms were counted. To analyze reproduction, three nematodes were placed onto a new OP50 seeded NGM plate and incubated for 48 h at 25 °C to count the eggs under a microscope. Body length was measured in a photo taken under the microscope by Motic Images Plus 3.0 software. Six replicates (*n* = 6) were conducted.

### Anticancer Study

The ISO-10993 standard recommendations were followed and the non-toxic samples were put into a tube with DMEM (Biowest SAS, France) without FBS, covering the whole surface area. The samples were incubated in humidified 5% CO_2_/95% ambient air for 72 h at 37 °C and used immediately.

A cell line of colon cancer (HT-29) and melanoma (B16)^11−13^ DMEM with 10% FBS was used to grow all the cells, along with 1% l-glutamine and 1% penicillin/streptomycin (Thermo Scientific Hyclone, Logan, UT, USA) at 37 °C and 5% CO_2_. The MTS assay was used to study the effect of the treatment on the cells, which were planted at 1 × 10^5^ cells/well onto a 96-well plate and incubated for 24 h at 37 °C. Film extracts (100 μL) replaced the medium in each well. Three serial dilutions of each extract were made. For positive control, 100 μL of the same medium was used to produce the film extracts that replaced the medium. After incubating the cells in humidified 5% CO_2_/95% ambient air for 24 h at 37 °C, 20 μL MTS solution was added to each well and incubated at 37 °C for 4 h. The plate was then analyzed at 490 nm (Flurostar Omega plate reader, BMG Labtech, Aylesbury, UK).

### Antibacterial Activity

We followed the agar disk diffusion protocol to test the antibacterial activity of the samples.^14^ The bacteria used was^16^ MRSE, RP62A.^15^ From a resuspended tryptic soy broth (TSB, Liofilchem) of *S. epidermidis* of about 10^8^ colony-forming units per milliliter (CFU/mL), lawns of this bacteria were prepared on a tryptic soy agar (TSA, Liofilchem) plates. The disks were carefully placed on the lawns of each bacteria and incubated at 37 °C for 24 h. The diameter of the inhibition zone (*d*_iz_) and sample disks were measured (*d*) by a digital caliper (ACHA, Spain). The inhibitory action of the materials on bacteria growth was tested by applying eq 2,^16^ in which the normalized antibacterial “halo” (*nw*_halo_) of each sample was calculated:
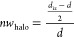
2

Each antibacterial test was performed three times on different days to analyze reproducibility.

### Antiviral Tests with the Enveloped Bacteriophage Φ6

*Bacterial growth ofPseudomonas syringae* (*P. syringae*, DSM 21482) was carried out first on a TSA (Liofilchem) and then in TSB (Liofilchem) at 25 °C and 120 rpm. The enveloped bacteriophage Φ6 (DSM 21518) was propagated. *P. syringae* and bacteriophage Φ6 were obtained from the Leibniz Institute DSMZ-German Collection of Microorganisms and Cell Cultures GmbH (Braunschweig, Germany).

A bacteriophage suspension (50 μL) at about 1 × 10^6^ PFU/mL (plaque-forming unit per mL) was added to each disk and incubated for 24 h. The samples were then placed in 10 mL TSB and subjected to sonication for 5 min at 25 °C and vortexing for 45 s. For the bacteriophage titration, serial dilutions were made of each sample. A mix of 100 μL of each bacteriophage dilution and 100 μL of the bacteria at OD_600nm_ = 0.5 was then made. The double-layer method was used to determine the infective activity of the virus.^17^ Top agar (4 mL; TSB + 0.75% bacteriological agar, Scharlau) with 1 mM CaCl_2_ was mixed with the bacteriophage-bacteria suspension to be poured onto TSA plates. The plates were incubated in an oven at a temperature of 25 °C for 24 h. Bacteriophage mixed (50 μL) with the bacteria without contact with any of the samples was used as the control. The bacteriophage titer of each type of sample was determined in PFU/mL to be compared with the control. The antiviral tests were performed three times on two different days (*n* = 6) to ensure reproducibility.

### Antiviral Tests on Non-Enveloped Bacteriophage MS2

*E. coli* (DSM 5695) and bacteriophage MS2 (DSM 13767) were obtained from the Leibniz Institute DSMZ-German Collection of Microorganisms and Cell Cultures GmbH (Braunschweig, Germany). *E. coli* was grown in TSA and subsequently in TSB at 37 °C and a speed of 240 rpm. Bacteriophage propagation of the MS2 bacteriophage MS2 was performed according to the Leibniz Institute DSMZ-German Collection of Microorganisms and Cell Cultures GmbH specifications. A bacteriophage suspension volume of 50 μL of bacteriophage with a titer of about 1 × 10^6^ PFU/mL was added to each sample and incubated for 24 h. The control sample consisted of 50 μL of the bacteriophage suspension that had not been in contact with the samples. Each sample was added to 10 mL of TSB and sonicated at 37 °C for 5 min, followed by 45 s of vortexing. Serial dilutions were released from each sample. Bacteriophage dilution (100 μL) was mixed with 100 μL of a host strain at OD_600nm_ = 0.2. Top agar (4 mL; TSB + 0.75% bacteriological agar) from Scharlau (Ferrosa, Barcelona, Spain) with 1 mM calcium chloride was mixed with the bacteriophage/bacteria suspension and poured onto TSA plates to be incubated for 24 h at 37 °C. Antiviral capacity was determined at 24 h of contact by calculating the bacteriophage titers in log (PFU/mL) for comparative analysis with the control sample. The antiviral assays were performed in triplicate on two different days (*n* = 6) to ensure reliable results.

### Double-Stranded RNA Extraction and Quantification

Double-stranded RNA extraction and quantification of the bacteriophage Φ6 were carried out to test whether or not viral particles remain attached to the Zn0, Zn0.1, Zn0.01, and Zn0.001 sample films and compared to the control before the antiviral assays to avoid false results. The bacteriophage solution (50 μL) at 1 × 10^6^ PFU/mL was dispersed on the disks and incubated for 30 min at 25 °C. The bacteriophage solution (50 μL) that had not been in contact with the samples (control) was incubated in the same conditions. Disks with bacteriophage solution and control were placed in a tube with 10 mL of TSB, sonicated for 5 min, and vortexed for 45 s, as in the antiviral assay. RNA was extracted according to the RNA extraction protocol provided by the Norgen Biotek Corp. (Ontario, Canada).^18^ First, a viral particle-lysing was carried out, followed by viral RNA purification. A nanodrop (Thermo Scientific, Waltham, USA) was used to quantify the RNA present in the sample films, and the results were expressed in ng/μL. These measurements were performed in triplicate to analyze reproducibility.

### Statistical Analysis

The statistical analysis consisted of a one-way analysis of variance followed by Tukey’s posthoc test (*p > 0.05, ***p > 0.001) on GraphPad Prism 8 software.

## Results

### Water Absorption

**Figure 1:**
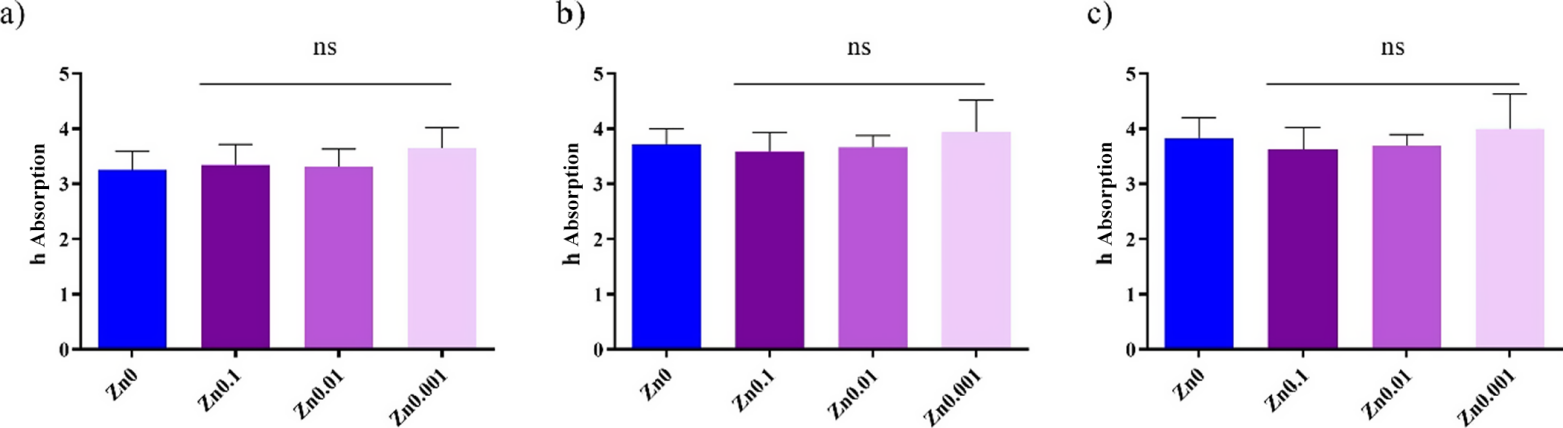
*h* absorption of liquid water ((*m*_hydrated film_ – *m*_dry film_)/*m*_dry film_) from alginate film crosslinked with Ca^2+^ (Zn0) and the films crosslinked with Ca^2+^ and Zn^2+^(Zn0.1, Zn0.01, and Zn0.001) after (a) 30 min, (b) 5 h, and (c) 24 h at 37 °C. The results of the statistical analysis of untreated film are indicated in the graph; n.s., not significant.

After drying the films at 60 °C for 48 h to constant weight, they were placed in 100 mL of distilled water in an oven at body temperature (37 °C). The films were weighed at three different times (30 min, 5 h, and 24 h) to calculate the *h* absorption, defined as the mass of the water divided by the mass of the dry film (Figure 1).

The h absorption of liquid water at 37 °C showed no statistically significant differences between the film crosslinked with Ca^2+^ (Zn0) and the films crosslinked with both Ca^2+^ and Zn^2+^ (Zn0.1, Zn0.01, and Zn0.001) after 30 min, 5 h, and 24 h.

### Zinc Release

**Figure 2:**
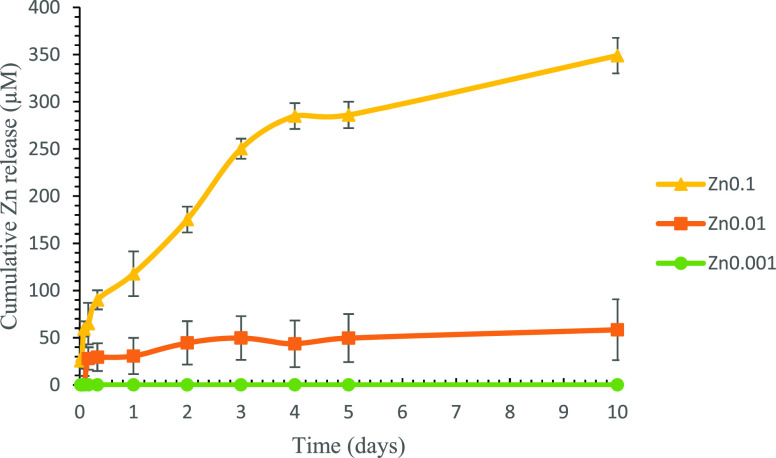
Zn release (μM) from the zinc films (Zn0.1, Zn0.01, and Zn0.001) after immersion in MQ water at 37 °C for 10 days.

The Zincon assay was used to study the cumulative Zn release profiles of the different Zn films after immersion in MQ water at 37 °C for 10 days (Figure 2).

The Zn0.1 film showed that zinc begins to be released at 30 min, while the Zn0.01 film indicated that zinc started to be released at 4 h. The amount of zinc released in the Zn0.001 film was negligible.

### Morphology and EDX Analysis

**Figure 3:**
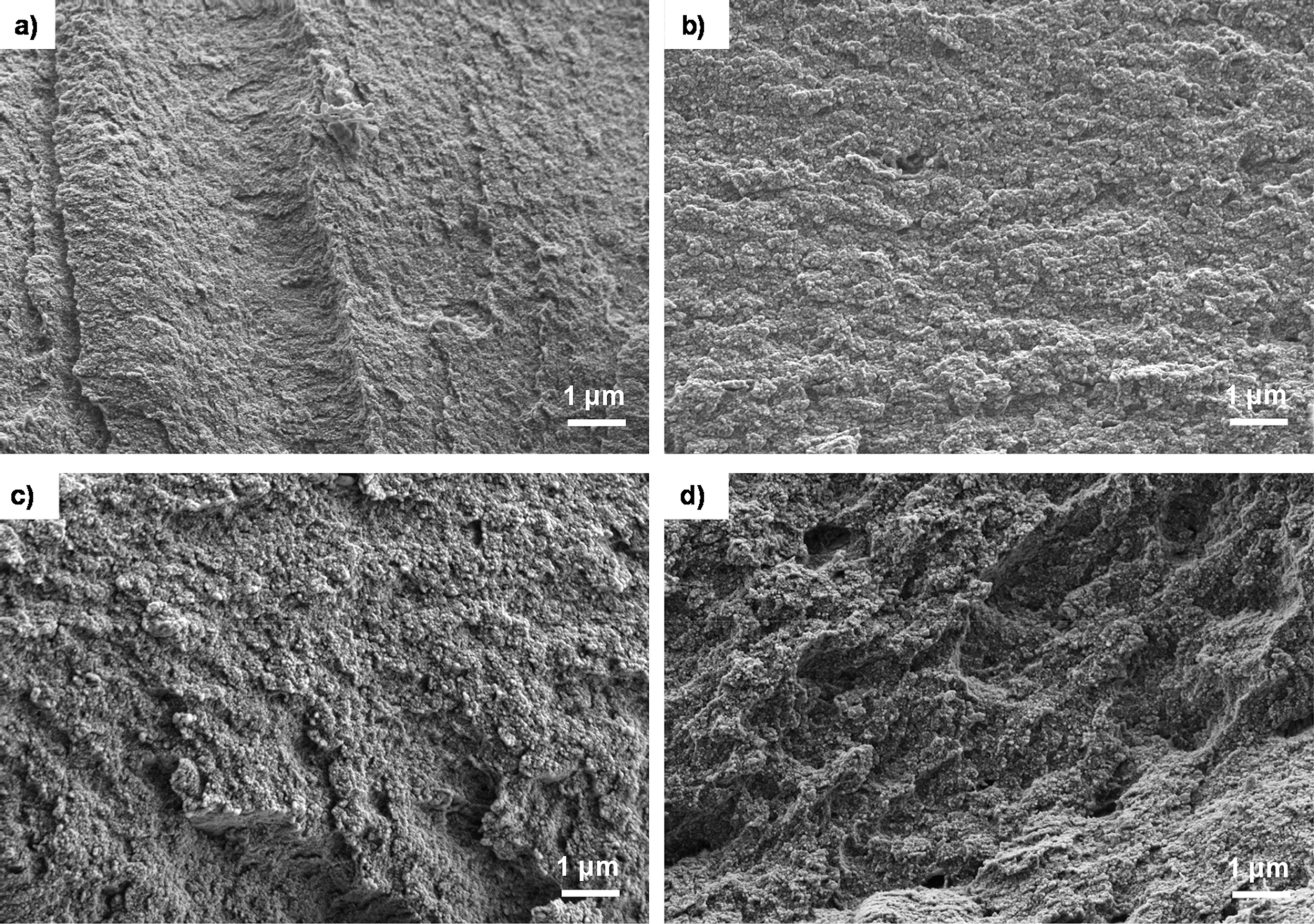
High-resolution FESEM cross-section microphotograph of the alginate hydrogels crosslinked with Ca or Ca–Zn ions. (a) Zn0, (b) Zn 0.001, (c) Zn 0.01, and (d) Zn 0.1. Magnification (a–d): 10 k×,

Figure 3 shows the FESEM images of the alginate hydrogels with different Ca–Zn contents. Calcium-crosslinked hydrogels (Figure 3a) show a quite smooth morphology, however, the hydrogels co-crosslinked with Ca^2+^ and Zn^2+^ (Figure 3b–d) show a rough morphology, more pronounced as the Zn^2+^ content increases. This observation can be related to the cooperative interaction between metallic cations and mannuronic (M) and guluronic (G) sequences within the structure of alginate.^19^ Ca^2+^ binds to the G blocks and mannuronic-guluronic blocks (MG) containing alternating M and G residues, while Zn^2+^ binds to M, G, and MG blocks,^20,21^ which results in morphological changes in the hydrogels, as reported previously.^19^ It is worth noting that the sample with the lower amount of Zn in the crosslinked solution (Zn0.001, Figure 3b) shows small morphological changes, more visible in samples Zn0.01 and Zn0.1 (Figure 3c,d), which were prepared with a higher concentration of Zn in the initial solution.

In order to confirm the presence of Ca and Zn ions, the crosslinked hydrogels were analyzed by EDX (Table 1). Although the EDX technique is semi-quantitative, the results indicate the presence of Ca^+2^ and Zn^2+^ in samples Zn0.01 and Zn0.1, in which 1.57 and 6.25% of the crosslinking ions were Zn ions. Even though the presence of Zn^2+^ could not be verified in sample Zn0.001 because its concentration was below the detection limit of EDX, the results confirm that higher levels of Zn^2+^ ions were progressively incorporated in the hydrogels as the Zn^2+^ concentration increased in the initial crosslinking solution.

**Table 1:** EDX Analysis of the Alginate Hydrogels^a^

sample	Ca (wt %)	Zn (wt %)
Zn0	100	0
Zn0.001	100	(*)
Zn0.01	98.43	1.57
Zn0.1	93.75	6.25

a (*) Zn^2+^ was below the EDX detection limit.

### FTIR

**Figure 4:**
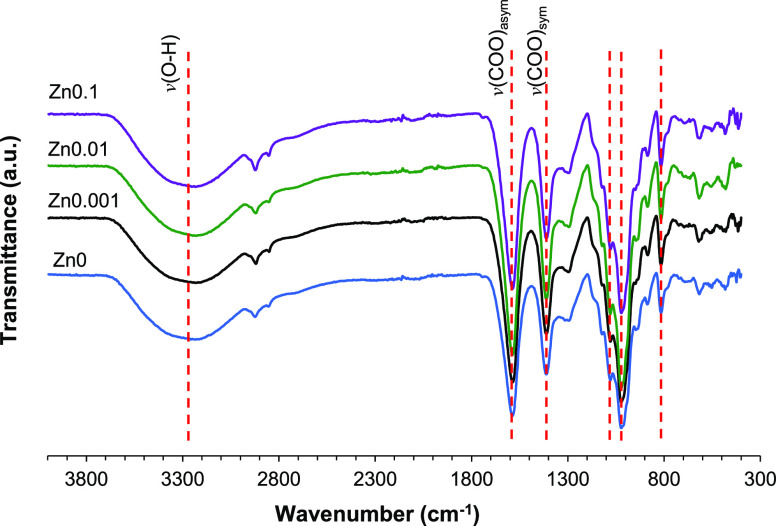
FTIR spectra in the region 4000–300 cm^–1^ of the alginate hydrogels (Zn0, Zn0.001, Zn0.01, and Zn0.1.

FTIR spectra of the different hydrogels are shown in Figure 4. The spectrum of calcium alginate (Zn0 sample) shows the characteristic peaks at 820 cm^–1^ related to CH bending, 1023 cm^–1^ related to CCH and COH bending, and 1079 cm^–1^ associated with CO and CCC stretching. The peaks at 1412 and 1587 cm^–1^ can be ascribed to the stretching vibration of the COO carboxylate groups (symmetric and asymmetric, respectively), and the wide peak between 3000 and 3500 cm^–1^ is related to OH stretching vibration.^20,22^ As expected, alginate hydrogels crosslinked with both Ca^2+^ and Zn^2+^ show the location of the peak at similar positions, although some peaks are slightly shifted due to the different binding character of Ca and Zn ions, as reported previously.^20,23^ Thus, no displacement was found in the band 3000–3500 cm^–1^ and the peak at 820 cm^–1^. However, a slight shift to lower wavenumber was found in the stretching vibration of symmetric carboxylate groups (1410 cm^–1^) and CCH and COH bending (1021 cm^–1^) in samples Zn0.01 and Zn0.1, respectively, which were prepared with higher amount of Zn in the crosslinking solution. This displacement may be related to the different binding of Ca and Zn to the M, G, and MG blocks of the alginate chains.^20,22^

### Toxicological and Proliferation Study

**Figure 5:**
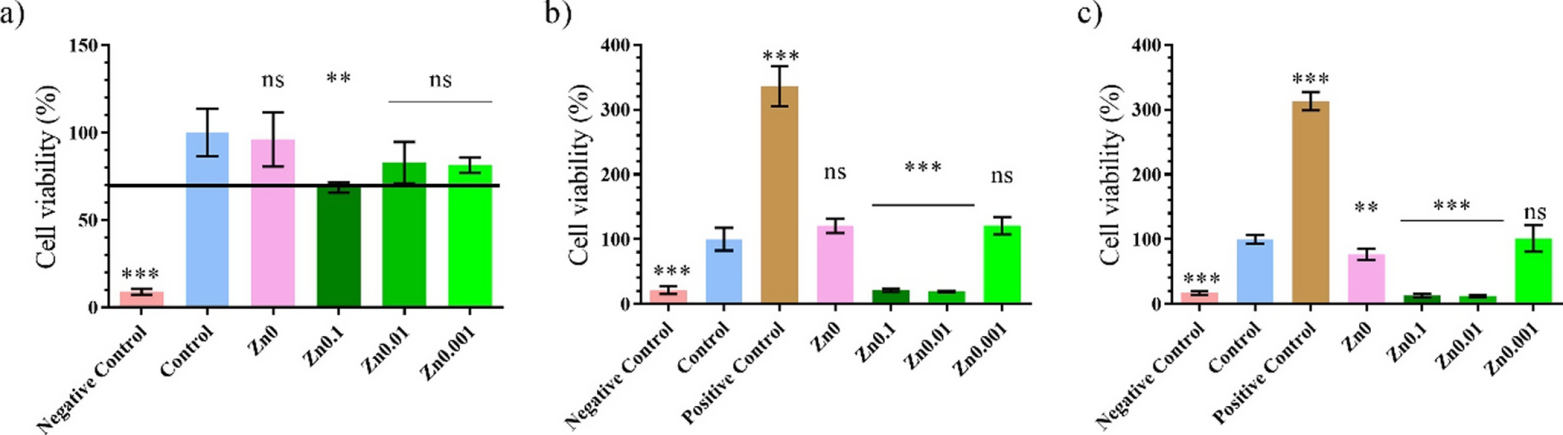
3-[4,5-dimethylthiazol-2-yl]-2,5-diphenyl tetrazolium bromide (MTT) cytotoxicity assay of extracts obtained from, untreated film (Zn0), film treated with different weights of zinc (Zn0.1, Zn0.01, and Zn0.001), control, positive, and negative controls cultured in human keratinocyte HaCaT cells for (a) 24 h, (b) 72 h, and (c) 96 h at 37 °C. **p < 0.01; ***p < 0.001; ns, not significant.

The MTT study was performed to study the viability of human keratinocyte HaCaT cells in the presence of the film extracts for 24, 72, and 96 h (Figure 5). The extract of the Zn0, Zn0.01, and Zn0.001 films after 24 h exposure (Figure 5a) showed no statistically significant differences in cell viability (%) to that of the control. Although the Zn0.1 sample did show statistically significant differences, the cell viability of the Zn0.1 extract was about 70%, which indicates that the samples are not cytotoxic according to the ISO-10993 standard.

The proliferative activity of films in the keratinocytes cell line was studied with the Zn0, Zn0.1, Zn0.01, and Zn0.001 films at longer times (72 or 96 h) (Figure 5a,b, respectively). Only the cells treated with the extracts of Zn0 and Zn0.001 films showed no statistically significant differences in cell viability to the control sample. The Zn0.1 and Zn0.01 films became toxic after 72 or 96 h. Even though some studies have reported that zinc can induce cell proliferation,^9^ the Zn0.001 film did not have this effect on human keratinocyte HaCaT cells compared to the growth factor (positive control), in good agreement with a previous study performed with zinc chloride.^8^

### In Vivo Toxicity Tests

**Figure 6:**
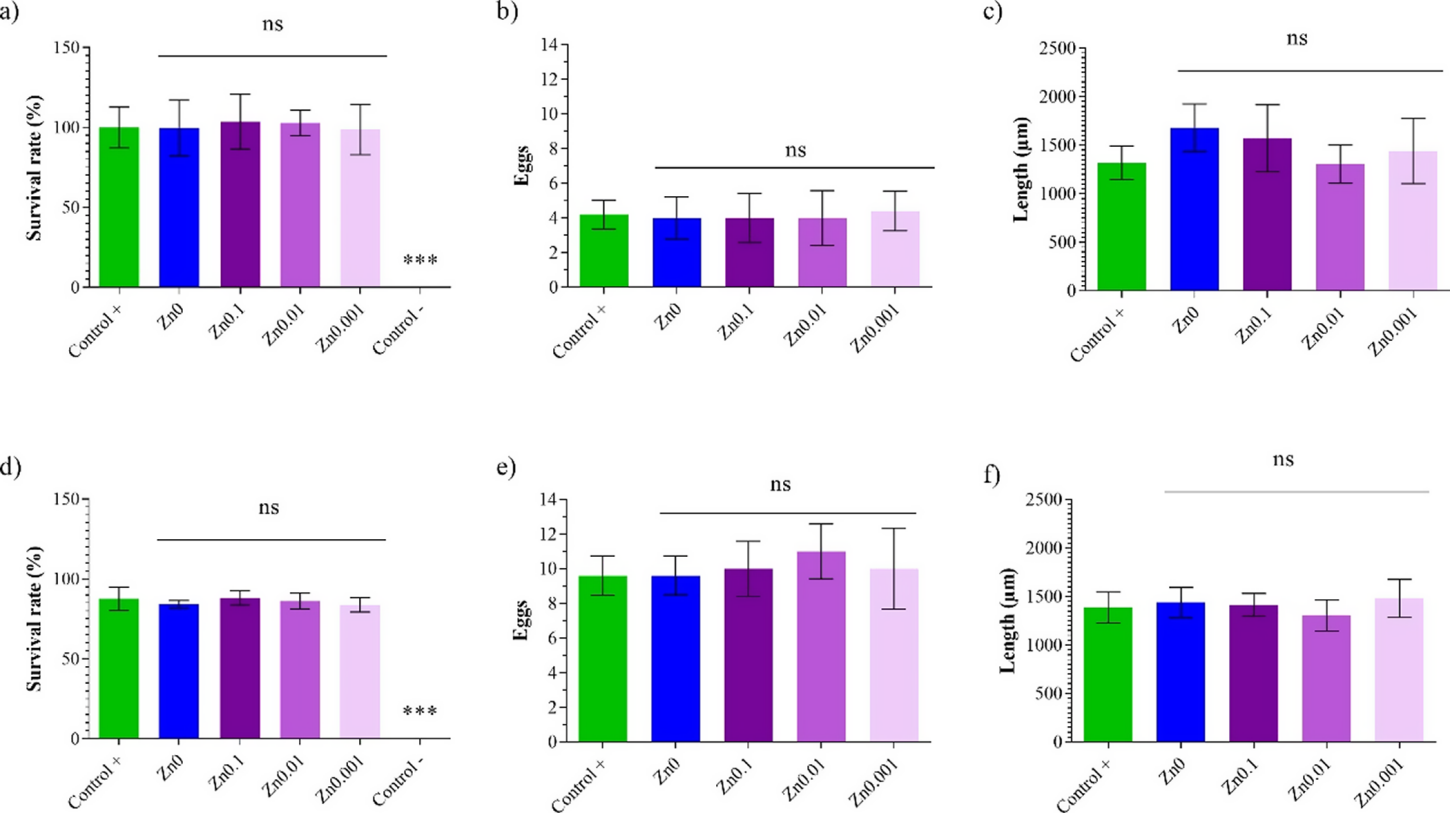
Measured parameters of the *Caenorhabditis elegans* in vivo toxicity model after 24 h of exposure to hydrogel extracts (acute toxicity): (a) for survival rate, (b) reproduction, and (c) body length; and 72 h (chronic toxicity): (d) survival rate, (e) reproduction, and (f) body length. Data (*n* = 6) as mean ± standard deviation. The results of the statistical analysis with respect to positive control are shown in the graph: ***p < 0.001; n.s, not significant.

The characteristics of the *C. elegans* model make it an ideal living system for the analysis of its survival against exposure to specific materials or particles. Toxicity classification of *C. elegans* has been shown to be as good a predictor as rat or mouse LD50.^24^*C. elegans* possesses human proteins, genes, lipids, and signaling ways and its digestive system has many characteristics similar to those of mammals.^25−29^ The genomics of this nematode is used to study human development and disease,^30^ and it does not involve important ethical problems. In many cases, conserved toxic mechanisms of action have been found between the nematode and mammals, apart from the fact that it has genes for most of the molecular components of the vertebrate brain. These consistent correlations make it possible to include trials using this model for early safety testing and as an integrated or staggered toxicity testing strategy, allowing the addition of an intermediate stage between in vivo studies and clinical trials.^30^ The different parameters of the nematodes were thus analyzed after an exposure of 24 h (acute toxicity) to the hydrogel extracts for the survival rate (Figure 6a), reproduction (Figure 6b), and body length (Figure 6c) and 72 h (chronic toxicity) survival rate (Figure 6d), reproduction (Figure 6e), and body length (Figure 6f).

The extract of the zinc films after 24 h exposure (acute toxicity) showed no significant differences with respect to the positive control sample (Figure 6a). After 72 h exposure (chronic toxicity), the Zn0, Zn0.1, Zn0.01, and Zn0.001 film extracts showed no significant differences with respect to the positive control sample (Figure 6d). Body length was the second parameter analyzed to determine the toxicity of the zinc film extracts. Growth was measured by body length in a picture taken under a microscope with Motic Images Plus 3.0 software. The results showed no significant difference between the Zn0, Zn0.1, Zn0.01, and Zn0.001 extracts with respect to the control after 24 h (Figure 6c) and 72 h (Figure 6f) of exposure. Reproduction was the last parameter analyzed to determine the toxicity of the zinc hydrogel extracts, for which three nematodes were placed on a new plate and incubated for 48 h. The eggs were then counted under a microscope. After 24 h of exposure (Figure 6b), the results showed no significant differences between the zinc film extracts with respect to the control. After 72 h of exposure (Figure 6e), the Zn0, Zn0.1, Zn0.01, and Zn0.001 hydrogels showed no significant difference with respect to the control sample.

### Anticancer Study

**Figure 7:**
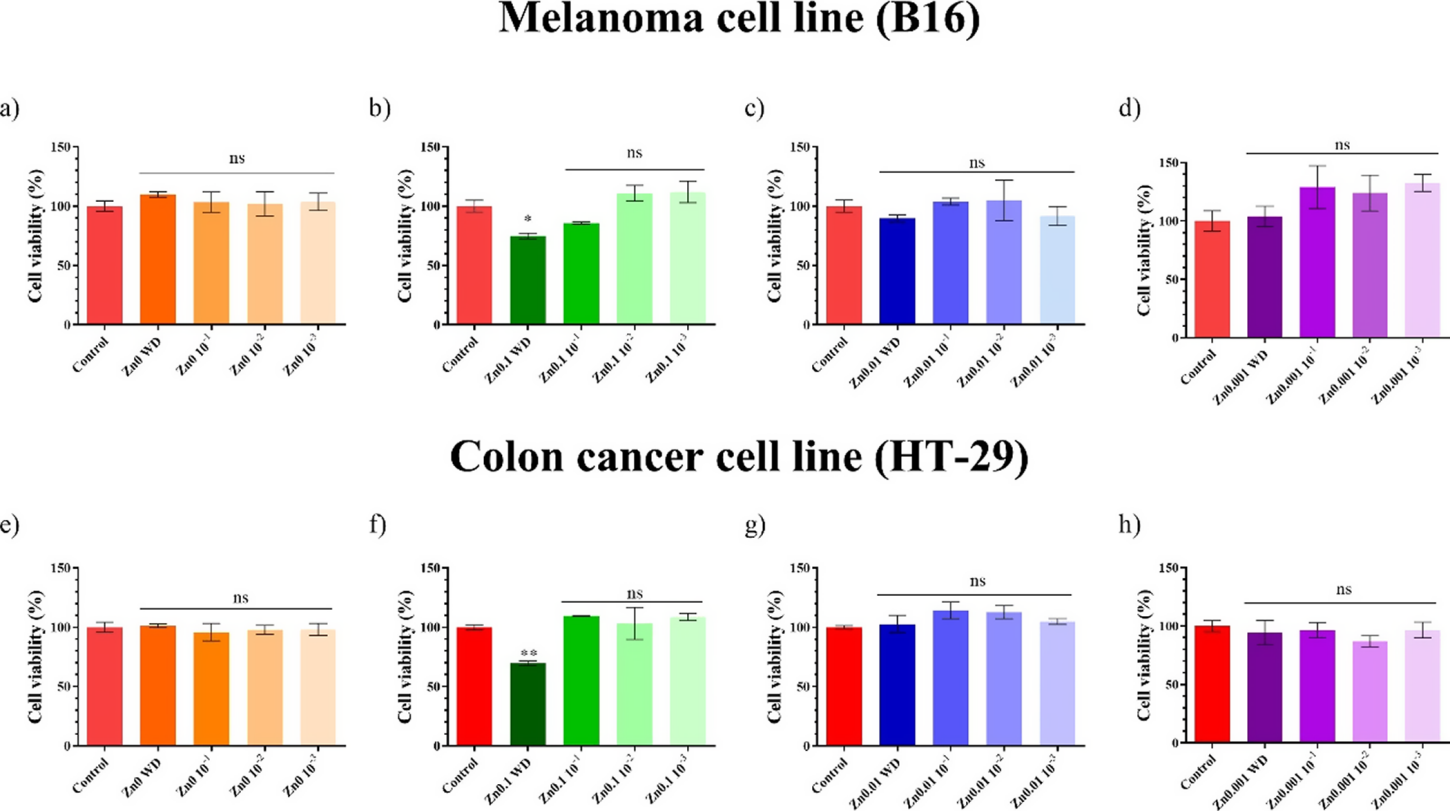
Anticancer activity of extracts of Zn films in melanoma (B16) cells treated with (a) Zn0, (b) Zn0.1, (c) Zn0.01, and (d) Zn0.001 extracts for 24 h. Anticancer activity of extracts of Zn films in colon cancer (HT-29) cells treated with the (e) Zn0, (f) Zn0.1, (g) Zn0.01, and (h) Zn0.001 concentration for 24 h. The results of the extracts are shown without dilution (WD), diluted at 10^–1^, 10^–2^, and 10^–3^. Data (*n* = 6) are expressed as mean ± standard deviation. The results of the statistical analysis with respect to the control are shown in the graph: *p < 0.05; **p < 0.01; n.s, not significant.

Melanoma cells (B16) were treated with serial dilutions of the Zn0, Zn0.1, Zn0.01, and Zn0.001 sample extracts for 24 h (Figure 7a–d). The extract of the Zn0 film showed no significant difference with respect to the control, as expected. On the other hand, the undiluted extract of Zn0.1 did show significant cell viability differences (less than 70%) with the control. The extract of Zn0.01 and Zn0.001 showed no significant differences with respect to the control sample.

Colon cancer cells (HT-29) were treated with serial dilutions of the Zn0, Zn0.1, Zn0.01, and Zn0.001 extract samples for 24 h (Figure 7e–h). The Zn0 film showed no significant difference with respect to the control sample, as expected, while the extract of the Zn0.1 film without dilution did show significant cell viability differences (less than 70%) with respect to the control. The extracts of Zn0.01 and Zn0.001 films showed no significant differences with respect to the control.

Zn0.1 was thus seen to manifest anticancer properties against melanoma and colon cancer cells.

### Antimicrobial Activity

**Figure 8:**
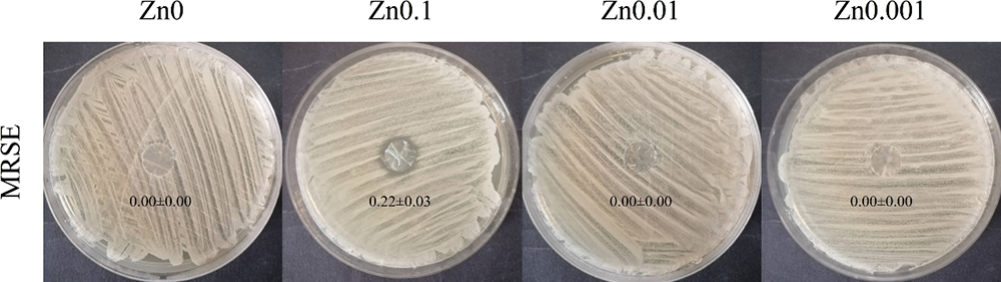
Antimicrobial agar disk diffusion tests with MRSE, Zn0, Zn0.1, Zn0.01, and Zn0.001 films after incubation at 37 °C. The normalized widths of the antibacterial halos, expressed as mean ± standard deviation and calculated with eq 2, are shown in each image.

The disk diffusion test was performed to study the antimicrobial activity of the sample films against Gram-positive multidrug-resistant bacteria (MRSE) (Figure 8).

The agar disk diffusion test showed that Zn0.1 possessed antibacterial activity against MRSE. The other samples (Zn0, Zn0.01, and Zn0.001) showed no bacterial inhibition zone. The bacterial cell envelope of MRSE seems to be susceptible to being destroyed by the zinc cations.

### Antiviral Test Using Enveloped Bacteriophage Φ6 and Non-Enveloped Bacteriophage MS2

**Figure 9:**
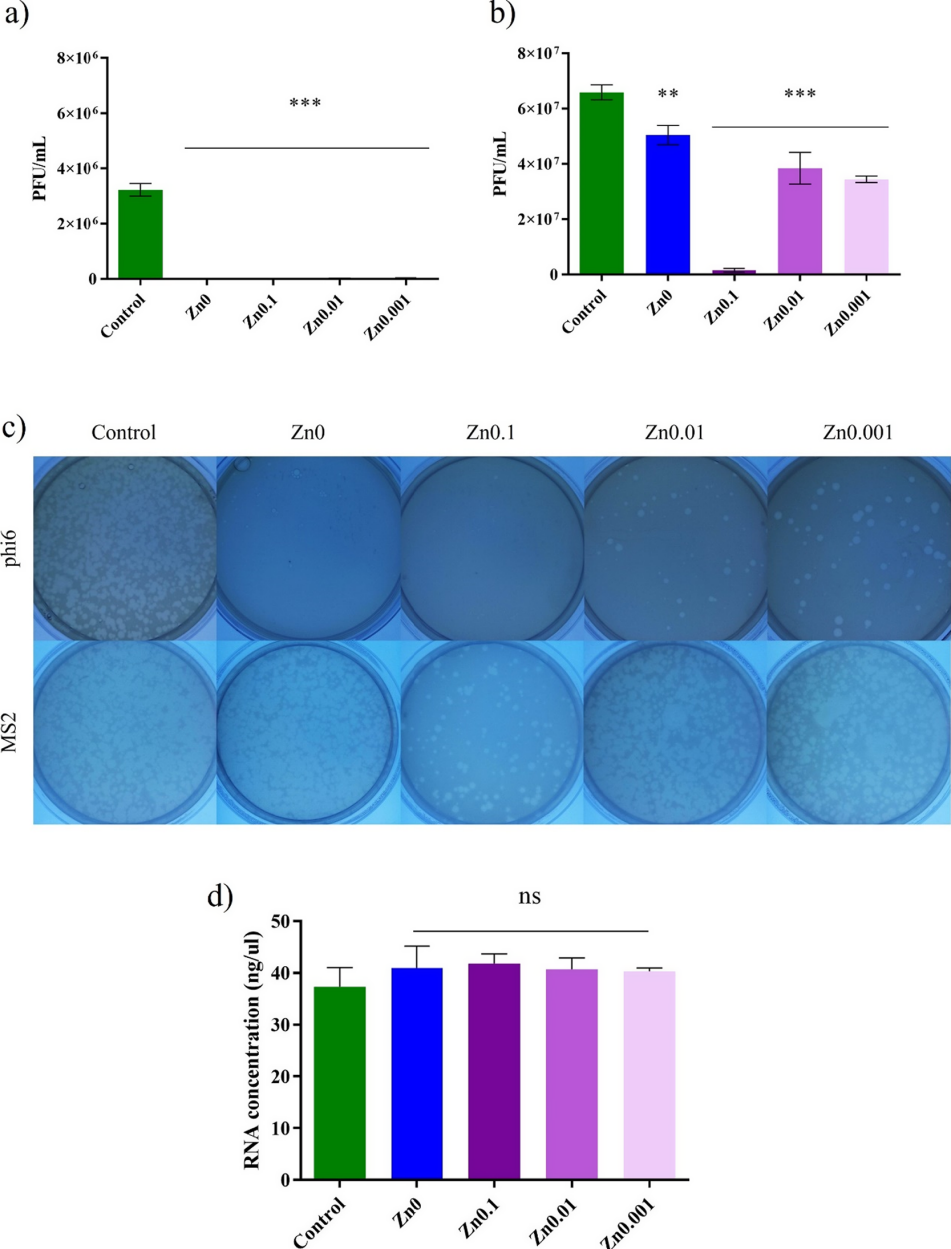
Reduction of infection titers of (a) bacteriophage phi 6 and (b) bacteriophage MS2 in plaque-forming units per mL (PFU/mL) measured by the double-layer method. Control, untreated film (Zn0), and film treated with different weights of zinc (Zn0.1, Zn0.01, and Zn0.001) at 24 h of viral contact. (c) Loss of bacteriophage phi 6 and MS2 viability measured by the double-layer method. Bacteriophage phi 6 titration images of undiluted samples and bacteriophage MS2 titration images of diluted 1/100 samples for control, alginate film crosslinked with Ca^2+^ (Zn0) and the films crosslinked with Ca^2+^ and Zn^2+^ (Zn0.1, Zn0.01, and Zn0.001) at 24 h of viral contact. (d) RNA concentration (ng/μL) of bacteriophage Φ6 measured in the control (without being in contact with the samples) and the same amount of bacteriophage after being in contact with the Zn0, Zn0.1, Zn0.01, and Zn0.001 films for 10 min; ***p < 0.001; **p < 0.01; ns, not significant.

Sonication and vortexing were performed to release all the viral particles after being in contact with the films. The plaque-forming units per mL (PFU/mL) of bacteriophage Φ6 and bacteriophage MS2 after being in contact for 24 h with the Zn0, Zn0.1, Zn0.01, and Zn0.001 are shown and compared with the control in Figure 9a and Figure 7b, respectively.

The results of the antiviral tests showed that the Zn0, Zn0.1, Zn0.01, and Zn0.001 film extracts possessed antiviral activity at 24 h of viral contact against enveloped bacteriophage Φ6 (Figure 9a). The results of the antiviral assays against bacteriophage MS2 showed that only the Zn0.1 sample had antiviral capacity for the same viral contact time (Figure 9b). After 24 h of viral contact between phi6 bacteriophage and the different films, bacterial lawns grew in the plate with few plaques (Figure 9c).

In contrast, only Zn0.1 showed a reduction in plaque-forming units against bacteriophage MS2 (Figure 9c). The antiviral capacity against enveloped bacteriophage of zinc-treated calcium-crosslinked alginate hydrogel was quite similar to the antiviral capacity of the calcium-crosslinked alginate hydrogel alone.^4^ At 24 h of viral contact with the bacteriophage Φ6, the % inactivation of the virus was 100% in Zn0 and Zn0.1. The films treated with the low quantity of zinc (Zn0.01 and Zn0.001) were 99.48 and 98.86% inactive, respectively (Table 2).

**Table 2:** Infection Titers Determined by the Double-Layer Method for the Antiviral Tests Performed with Bacteriophage Φ6 and Bacteriophage MS2 Shown as Mean ± Standard Deviation, Percentage of Viral Inactivation and Log(PFU/mL) Reduction with Respect to Control and after Being in Contact with the Untreated Film (Zn0), and the Film Treated with Zinc (Zn0.1, Zn0.01, and Zn0.001) for 24 h

	control	Zn0	Zn0.1	Zn0.01	Zn0.001
bacteriophage Φ6 (PFU/mL)	3.23 × 10^6^ ± 2.32 × 10^5^	0.00 ± 0.00	0.00 ± 0.00	1.67 × 10^4^ ± 1.42 × 10^4^	3.67 × 10^4^ ± 5.77 × 10^3^
log reduction		6.51	6.51	2.42	1.95
% inactivation		100.00	100.00	99.48	98.86
bacteriophage MS2 (PFU/mL)	6.59 × 10^7^ ± 2.72 × 10^6^	5.04 × 10^7^ ± 3.47 × 10^6^	1.55 × 10^6^ ± 6.69 × 10^5^	3.48 × 10^7^ ± 5.74 × 10^6^	3.45 × 10^7^ ± 1.15 × 10^6^
log reduction		≈0	1.65	0.24	0.28
% inactivation		≈0	97.65	≈0	≈0

In the case of bacteriophage MS2 at 24 h of viral contact, the % inactivation of the virus was 97.65% with Zn0.1, although the % inactivation of the virus was negligible (≈0) with the other sample films, within the experimental uncertainty.

### Double-Stranded RNA Extraction and Quantification

RNA extraction and quantification of bacteriophage Φ6 after being in contact with the Zn0, Zn0.1, Zn0.01, and Zn0.001 films were carried out to show that the virus did not remain adhered to the surface of the film before the antiviral assays, which could have given false results. The amount of RNA showed no significant differences between the control and after the virus had been in contact with the different samples (Figure 9d).

## Conclusions

Alginate hydrogels were synthesized by crosslinking sodium alginate with calcium cations and different amounts of zinc chloride. The physical and biological properties of these hydrogels were studied in terms of water sorption, zinc release, anticancer, antiviral, antibacterial, and in vitro and in vivo toxicity. The calcium alginate hydrogel film crosslinked with the highest amount of zinc showed similar water sorption properties to calcium alginate and released a suitable amount of zinc to provide anticancer activity against melanoma and colon cancer cells, antibacterial properties against MRSE, one of the most important bacteria resistant to antibiotics, and antiviral activity against enveloped and non-enveloped viruses. This film showed no toxic effect in vitro in keratinocyte HaCaT cells and in vivo in the *C. elegans* model and therefore holds great promise for biomedical applications that require biocompatible materials with antimicrobial and anticancer activity.
